# *Nocardia thailandica* Brain Abscess in an Immunocompromised Patient

**DOI:** 10.1155/2021/6620049

**Published:** 2021-06-09

**Authors:** Muhammad Effendi, Samad Tirmizi, Dayna McManus, Anita J. Huttner, David R. Peaper, Jeffrey E. Topal

**Affiliations:** ^1^Department of Pharmacy Practice and Administration, Ernest Mario School of Pharmacy, Rutgers University, Piscataway, NJ, USA; ^2^Department of Pharmacy, Capital Health Regional Medical Center, Trenton, NJ, USA; ^3^Department of Pharmacy, Yale New Haven Hospital, New Haven, CT, USA; ^4^Department of Pathology, Yale School of Medicine, New Haven, CT, USA; ^5^Department of Laboratory Medicine, Yale School of Medicine, New Haven, CT, USA; ^6^Department of Internal Medicine, Section of Infectious Diseases, Yale School of Medicine, New Haven, CT, USA

## Abstract

**Objectives:**

Successful treatment for *Nocardia thailandica* is not well elucidated in the literature. To the best of our knowledge, *N. thailandica* has not yet been described in the medical literature to cause central nervous system (CNS) infection from brain abscess. We report the case of an immunocompromised patient who underwent successful treatment to treat his brain abscess caused by *N. thailandica*.

**Methods:**

After failing medical therapy, the patient underwent a craniotomy, and tissue was sent for culture. Upon identification by 16S rDNA sequencing, the organism causing infection was identified to be *N. thailandica*.

**Results:**

Based on susceptibilities, the patient was treated with IV ceftriaxone 2 grams daily for five months. The patient demonstrated clinical and radiological improvement which persisted to 7 months after initiation of therapy.

**Conclusions:**

To the best of our knowledge, this is the first documented case of a brain abscess due to *N. thailandica* which was successfully treated. Due to the location of the infection, ceftriaxone was chosen because of optimal CNS penetration. Ceftriaxone monotherapy demonstrated clinical and radiographic treatment success resulting in the successful treatment of this infection.

## 1. Introduction

*Nocardia* spp. are responsible for both localized infections, such as pneumonia, and disseminated infections which can occur in the central nervous system (CNS). Immunosuppression is a known risk factor for nocardia infection. *Nocardia thailandica* is a rare species which has only been documented to cause infections in humans four times in the medical literature since its original classification in 2004. Here, we report the first documented case of a brain abscess due to *N. thailandica* which was successfully treated with ceftriaxone monotherapy.

## 2. Case Presentation

A 44-year-old immunocompromised male presented to an outside hospital with new-onset seizures and altered mental status. Relevant past medical history included pure red cell aplasia, concomitant autoimmune hemolytic anemia, and T-cell large granular lymphocytic (LGL) leukemia for which he received chemotherapy with cladribine two years prior to presentation. A CT of the brain revealed a small ring-enhancing lesion (measuring 0.9 (AP) × 0.9 (TR) × 0.9 (CC) cm) in the right parietal lobe with surrounding edema. A lumbar puncture was performed which revealed 11 WBC (41% lymphocytes, 7% monocytes, 52% granulocytes), glucose of 83 mg/dL, and protein of 94 mg/dL. The routine cerebrospinal fluid (CSF) bacterial culture was negative for growth. The fungal smear was negative; however, the fungal CSF culture grew *Candida parapsilosis*. Based on these findings, he was initiated on fluconazole for treatment.

Since the patient did not exhibit clinical improvement following ten days of fluconazole therapy, a brain biopsy was performed of the right parietal lesion. All bacterial, fungal, and acid-fast bacilli smears and cultures from the biopsy were negative. Tissue was sent for pathology, and upon initial review, it was believed that the organisms, which were positive by acid-Schiff (PAS) and Grocott Methenamine-Silver (GMS), had a size similar to *Cryptococcus* spp. Of note, serum cryptococcal antigen was negative. Consequently, the patient was empirically started on liposomal amphotericin B and flucytosine for presumed CNS cryptococcal infection. After an additional review by the pathologists, it was felt that these “microorganisms” were most consistent with corpora amylacea, not *Cryptococcus* spp. Additionally, postbiopsy MRI scan suggested that the lesion in question was not sampled during the biopsy. Consequently, the decision was made to continue the liposomal amphotericin B but discontinue the flucytosine. After four weeks of amphotericin B, a repeat MRI revealed mild improvement (3 mm reduction of ring-enhancing lesion); therefore, a decision was made to stop amphotericin therapy and start fluconazole therapy. A repeat MRI one week later revealed an increase in the size of the right parietal lobe lesion. Therefore, the patient was restarted on liposomal amphotericin B and flucytosine therapy.

**Figure 1:**
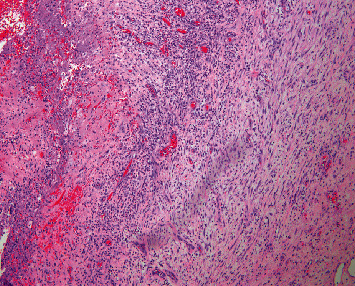
A representative image demonstrates necrosis with a surrounding rim of granulation tissue, composed of a dense polymorphous inflammatory infiltrate.

After receiving 53 days of combination antifungal therapy without clinical improvement, it was determined that the patient had failed medical therapy and surgical intervention was indicated. The patient was then transferred to our institution for craniotomy with incision and drainage of the right intraparietal lesion. A biopsy sample was sent to anatomic pathology for analysis. The tissue was stained with hematoxylin and eosin to demonstrate a cerebral abscess with features of encapsulation (see Figure 1). The abscess is composed of a necrotic center with a surrounding rim of granulation tissue, which contains a dense polymorphous inflammatory infiltrate composed of neutrophils, macrophages, lymphocytes, and plasma cells. A capsule with embedded fibroblasts and capillaries separates the abscess from the adjacent reactive brain. Cultures from this procedure revealed branching Gram-positive rods on Gram stain.

Given the concern for *Nocardia,* the patient was initiated on ceftriaxone and sulfamethoxazole/trimethoprim (TMP/SMX), in addition to metronidazole and amphotericin B. Once the patient was clinically stable, the patient was transferred back to the outside institution for further care. At the outside institution, ceftriaxone was discontinued and meropenem was initiated. Over the next two days, the patient developed severe headaches and profound left-sided weakness. A repeat CT of the brain revealed an interval increase in vasogenic edema involving the right frontoparietal, occipital, and temporal regions with the development of 5 mm midline shift. Given worsening of his clinical status and imaging, he was transferred back to our institution. The day following the transfer, the Gram-positive rods on Gram stain were identified to be *Nocardia thailandica* by 16S rDNA sequencing with a 99.8% match to the *N. thailandica* type strain IFM 10145.

Upon definitive identification, ceftriaxone 2 g IV daily was initiated, TMP/SMX was continued on a dose of 15 mg/kg/day of the trimethoprim component, and meropenem was discontinued. Metronidazole and amphotericin were not continued given the findings. Two weeks after the identification of *N. thailandica*, susceptibility testing at the University of Texas revealed susceptibility to ceftriaxone. Given the susceptibility results and the development of significant hyperkalemia, the decision was made to discontinue TMP/SMX after 19 total days of therapy.

Following the optimization of the antimicrobial regimen, the patient improved both clinically and radiographically. He was discharged from the hospital 63 days from the brain biopsy that revealed *N. thailandica*. The patient completed a total of five and a half months of ceftriaxone monotherapy.

MRI of the brain obtained four months after initiation of therapy revealed a stable associated mass effect and surrounding edema. A subsequent MRI obtained 7 months from the initiation of therapy revealed slightly decreased interval size of the surgical cavity and no significant interval change in vasogenic edema/gliosis. The patient did not experience any additional seizures or neurologic deficits after starting ceftriaxone therapy.

## 3. Discussion

*Nocardia* is a genus of the aerobic actinomycetes family, a large and diverse group of Gram-positive bacteria. Accurate and rapid identification of *Nocardia* spp. is critical to optimize empiric antimicrobial therapy. Routine identification of *Nocardia* to the species level is a time-consuming process. Furthermore, these phenotypic tests may be inconclusive and difficult to interpret, resulting in limitations in the identification of *Nocardia* species [1, 2]. Additionally, the genus *Nocardia* has undergone substantial taxonomic revisions with the advent of molecular methods, rendering interpretation of identification challenging compared to historic data [2]. Methods that do not rely upon differential growth characteristics including antibiotic profiles such as 16S rDNA sequencing and matrix-assisted laser desorption ionization-time of flight mass spectrometry (MALDI-TOF MS) can provide more rapid and accurate identifications of challenging organisms such as *Nocardia* spp [3].

*N. thailandica* was first identified in 2004 from a soft tissue infection. In that report, the authors did not provide information on antimicrobial therapy [4]. The next report of *N. thailandica* was by Reddy et al., who isolated twenty *Nocardia* spp. from ocular infections; *N. thailandica* represented just one of the twenty isolated species [5]. While this report gave more thorough information on the susceptibilities of the isolate, the patient's treatment regimen was not delineated.

Canterino et al. reported a 66-year-old patient on immunosuppressive therapy status post-lung transplant who had pulmonary nocardiosis [6]. Upon identification of *N. thailandica* via percutaneous lung biopsy, the patient was treated with meropenem for one month, followed by oral minocycline to complete six to twelve months of therapy. Following six weeks of antibiotic therapy, follow-up imaging revealed a good overall response to therapy.

Bourbour et al. reported a 53-year-old, immunocompetent man with chronic bronchitis, who presented with persistent fever and cough and was found to have nodular infiltrates on chest X-ray [7]. A bronchoalveolar lavage sample grew *Nocardia thailandica* and the patient was treated with TMP/SMX and linezolid for 6 months. The authors reported that the patient's symptoms resolved completely.

Optimal therapy for *Nocardia* spp. has not been well established [1, 8]. Considerations for selecting therapy should be based on species of *Nocardia* identified, site, and severity of infection. Combination therapy against *Nocardia* spp. has been thought to provide enhanced activity and is recommended for initial treatment for most forms of nocardiosis. Single-drug therapy may be sufficient after species identification, and antimicrobial drug susceptibility information can be confirmed [8]. As no randomized controlled trials provide guidance on optimal treatment, this recommendation is largely based on clinical experience. Patients with CNS nocardiosis may have increased mortality; therefore, combination therapy is often strongly recommended [9]. Ceftriaxone, meropenem/imipenem, sulfonamides, linezolid, and amikacin are often options for the treatment of nocardiosis. However, based on drug susceptibility testing at the species level, there is a wide range of variation in coverage [10, 11].  Table 1 reveals antibiotic susceptibility data of *Nocardia thailandica* from the literature and our case.

Of the four strains of *N. thailandica* with full susceptibility data reported, all were susceptible to ceftriaxone and carbapenems. Of the most common susceptible agents, ceftriaxone and TMP/SMX have the most optimal blood-brain barrier penetration and may be ideal for the treatment of CNS nocardiosis [12]. Due to poor penetration into the CNS and an unfavorable toxicity profile with prolonged administration, amikacin would be a suboptimal option. Linezolid, despite good CNS penetration, has significant adverse effects associated with prolonged use, such as thrombocytopenia, peripheral neuropathy, and optic neuropathy, the latter two of which are irreversible. Imipenem also penetrates the CNS well; however, it has well-known toxicity of seizures limiting its use. Moxifloxacin reaches high CSF concentrations and is active against selected *Nocardia spp*.; however, it has extremely limited human data [10, 12]. Meropenem, while certainly an option for CNS nocardiosis, may prove to be overly broad and increase the risk of selecting resistant organisms. Additionally, the increased dosing frequency of meropenem would also prove to be a limitation of outpatient treatment as compared to the dosing regimen selected for our patient, once-daily dosing of ceftriaxone. In summary, ceftriaxone and TMP/SMX would be potentially ideal agents for the treatment of CNS nocardiosis given their coverage for *Nocardia thailandia* and good penetration into the CNS.

## 4. Conclusion

This is the first documented case of a brain abscess due to *N. thailandica* which was successfully treated. Due to the location of the infection, ceftriaxone and TMP/SMX were chosen because of optimal CNS penetration. TMP/SMX was discontinued approximately three weeks after initiation due to hyperkalemia. Ceftriaxone monotherapy demonstrated clinical and radiographic treatment success resulting in the successful treatment of this infection.

## Figures and Tables

**Table 1:** Susceptibility profile of *Nocardia thailandica* based on the existing published literature.

	AMK	AZI	AMC	FEP	CRO	CIP	CLR	DOX	GAT	IPM	LZD	MIN	MER	MXF	TOB	SXT
Reddy et al. [5]	S	R	—	—	—	R	R	—	R	—	—	—	—	—	S	—
Schlaberg et al. [11]	S	—	R	—	S	R	S	—	—	S	S	R	—	S	S	S
Canterino et al. [6]	S	—	S	S	S	R	S	—	—	S	S	S	S	S	S	S
McTaggart et al. [10]	S	—	R	S	S	R	S	S	—	S	S	S	—	S	S	^*∗*^
Bourbour et al. [7]	S	—	—	—	—	R	—	—	—	—	S	—	—	—	—	S
Effendi 2021	S	—	R	—	S	R	S	I	—	S	S	I	—	I	S	S

AMK: amikacin; AMC: amoxicillin-clavulanic acid; AZI: azithromycin; FEP: cefepime; CRO: ceftriaxone; CIP: ciprofloxacin; CLR: clarithromycin; DOX: doxycycline; GAT: gatifloxacin; IPM, imipenem; LZD: linezolid; MIN: minocycline; MXF: moxifloxacin; TOB: tobramycin; SXT: trimethoprim-sulfamethoxazole. S: susceptible; I: intermediate; R: resistant; ^*∗*^1/2 strains were susceptible and the other resistant.

## Data Availability

The data used to support the findings of this study are available from the corresponding author upon request.
